# Characterization of ligand binding to dopamine receptors with fluorescence anisotropy based assay

**DOI:** 10.1186/2193-1801-4-S1-P1

**Published:** 2015-06-12

**Authors:** Anni Allikalt, Ago Rinken

**Affiliations:** Department of Bioorganic Chemistry, Institute of Chemistry, University of Tartu, Competence Centre on Health Technologies, Estonia

**Keywords:** G protein-coupled receptors, Fluorescence anisotropy, dopamine receptors

Dopamine receptors are G-protein-coupled receptors (GPCRs), which are involved in a wide variety of physiological processes. Abnormalities in dopaminergic signal transduction are associated with many different diseases. Therefore, dopamine receptors are targets for variety of drugs involved in disorders like schizophrenia, Parkinson’s disease, depression and many others. In order to develop drugs with less side effects and better efficacy it is necessary to understand and characterize receptor-ligand interactions in further detail. In addition, measuring the on- and off-rates of different ligands provides important information about the kinetic profiles of potential drug candidates. We have applied fluorescence anisotropy (FA) assay to investigate kinetic properties and affinities of different ligands for dopamine D1 receptor. For that we have implemented budded baculoviruses as a source of recombinant protein (Veiksina et al., 2014). As a result, we have seen that fluorescent ligand Bodipy-FL-SKF-83566 is suitable for the pharmacological characterization of non-labelled dopaminergic ligands. The obtained results are in good agreement with the data obtained from the radioligand [3H]SCH 23390 binding experiments with the same baculovirus preparations. In conclusion, by using fluorescence based detection assay, we are now able to perform real-time monitoring of ligand binding. Obtained results demonstrate that fluorescence anisotropy based assay is applicable for the study of dopamine receptors and their ligands.

